# Penalty 4-Node Quadrilateral Element Formulation for Axisymmetric Couple Stress Problems

**DOI:** 10.3390/ma17225486

**Published:** 2024-11-10

**Authors:** Yongkang Jiang, Yan Shang

**Affiliations:** State Key Laboratory of Mechanics and Control for Aerospace Structures, Nanjing University of Aeronautics and Astronautics, Nanjing 210016, China; jiangyongkang@nuaa.edu.cn

**Keywords:** FEM, axisymmetric, size effect, couple stress theory, penalty element

## Abstract

To address the issue of size effects in axisymmetric deformation of small-scale solids, this work proposes a 4-node 12-DOF element for axisymmetric problems based on the consistent couple stress theory (CCST), following the framework of the unsymmetric finite element method. With the use of the penalty function method, an independently assumed rotational field is introduced into the virtual work principle to approximate the physical rotation, ensuring the satisfaction of the *C*^1^ continuity requirement of the CCST in a weak form. As a benefit, the enriched *C*^0^ isoparametric-based interpolation is employed to construct the test functions for displacement and rotation. Furthermore, the force-stress field that satisfies the equilibrium equations related to axisymmetric deformation is employed as the element’s force-stress trial function. In order to circumvent locking issues, reduced integration is employed in the penalty stiffness integration process. The numerical results demonstrate that the new element exhibits high computational accuracy and convergence rate in both static and modal analysis problems, effectively capturing size-dependent phenomena.

## 1. Introduction

With the advancement of technology, the application of micro-nano structures has become increasingly widespread. A substantial body of experimental evidence accumulated over recent decades has demonstrated that the mechanical properties of micro-nano structures are closely correlated with the dimensions of the materials employed. For example, Fleck et al. [1] observed in their torsion experiments on fine copper wires that as the diameter of the wire decreased, its torsional stiffness increased significantly. Furthermore, micro-indentation tests have demonstrated that the indentation hardness also increases as the size decreases [2]. This phenomenon, whereby the mechanical properties of materials undergo a significant change when their dimensions are reduced to a certain extent, is known as the size effect. The absence of an internal length scale in the constitutive model precludes the classical continuum theory from predicting size-dependent deformations observed at small scales. A number of higher-order continuum theories have been proposed to describe this phenomenon, including the nonlocal theory [3,4], the micropolar theory [5,6], the couple stress theory [7,8,9,10,11,12], and the strain gradient theory [1,13,14]. Among these higher-order theories, the couple stress theory stands out for its concise mathematical expressions and explicit physical interpretations, which has contributed to extensive application.

The concept of couple stress in materials was initially proposed by Voigt [15], who put forth the idea that an object might be composed of very minute elements, thereby allowing for the existence of moment couples. The Cosserat brothers [7] introduced the couple stress theory by introducing a micro-rotation quantity, which is independent of the physical rotation of the body, and established a mathematical model for the analysis of the effects of couple stress. Then, researchers, including Mindlin and Tiersten [8], Toupin [9], and Koiter [10] built upon the Cosserat theory by assuming that micro-rotation and macro-rotation are equivalent, with the displacement field being the only independent field that also determines the rotation field, leading to the classical couple stress theory (TMK-CST) where the curvature tensor is the gradient of the rotation vector. In the case of linear elastic isotropic materials, the classical couple stress theory necessitates the introduction of two material length scale parameters, which correspond to the curvature tensor. Yang and colleagues [11] introduced the couple moment balance equation as an additional equation for couple stress, addressing the free-floating nature of the couple vector, which led to the modified couple stress theory (MCST) where the curvature is defined as the symmetric part of the physical rotation gradient. In the case of linear elastic isotropic materials, the modified couple stress theory necessitates the introduction of a single material length scale parameter, which is related to the symmetric curvature tensor. Hadjesfandiari et al. [12] pointed out that both the TMK-CST and the MCST have issues with the indeterminacy in the spherical part of the couple stress moment tensor and the inconsistency in the boundary conditions. The appearance of the indeterminacy of the spherical part of the couple stress moment tensor is troublesome in most cases, especially those with torsional deformation. In addition, it has been shown that both the TMK-CST and MCST fall short in accurately describing the pure bending deformation of plates [16]. The inconsistency in the MCST is particularly evident, as it is founded on torsion deformation rather than curvature bending. Consequently, the MCST predicts no couple stresses and no size-effects for the pure bending of a plate into a spherical shell.

Hadjesfandiari and colleagues [12] proposed the consistent couple stress theory (CCST) from a kinematic perspective using the principle of virtual work. This was achieved by considering the actual displacement and rotation of continuous bodies and incorporating allowable boundary conditions. It is reported that, the CCST overcomes the indeterminacy in the spherical part of the couple stress tensor, and the body couple load can be converted into corresponding body forces and surface forces, thereby not appearing in the equilibrium equations [12]. The curvature tensor is defined as the skew-symmetric part of the physical rotation gradient. In the case of linear elastic isotropic materials, the consistent couple stress theory necessitates the introduction of a single material length scale parameter associated with the skew-symmetric curvature tensor. It is challenging to ascertain with certainty which of the couple stress theories is more effective [17,18]. Despite the controversy, the CCST has seen an increasing application in recent years. For instance, Darrall et al. [19] proposed a mixed finite element variational formulation for analyzing the piezoelectric size-effect in centrosymmetric cubic and isotropic dielectric materials based on the CCST. Based on the CCST, Chakravarty et al. [20] proposed a penalty-based finite element framework for solving general problems of linear elastic isotropic materials under plane strain conditions. Guarín-Zapata et al. [21] established a finite element formulation for the wave propagation problems associated with the CCST under extended Bloch periodic boundary conditions.

For practical engineering problems with complex geometries and boundary conditions, it is extremely challenging to obtain analytical solutions using either classical continuum theory or higher-order continuum theories. The finite element method (FEM), as one of the most commonly used numerical methods in scientific research and engineering computations, offers an effective approach for solving such complex problems. The consistent couple stress theory involves force-stress and couple stress. Therefore, to obtain accurate and reliable reference solutions, a sufficiently large number of elements must be used [22]. In three-dimensional problems, once the mesh size reaches tens of thousands, the computation time can extend to several hours or even longer, leading to inefficiency and high computational costs. However, within the context of three-dimensional continuum mechanics, there exists a class of special problems characterized by axisymmetric in geometry, materials, and boundary conditions. Since all variables are independent of coordinate *θ*, any deformation in the *rz*-plane represents the deformation of the entire solid of revolution, thereby transforming a three-dimensional problem into a two-dimensional one with only two unknown variables, significantly reducing computational costs. For instance, Chen et al. [23] established patch test functions for axisymmetric elements under conventional stress and couple stress theories, concluding that the patch test functions cannot include non-zero constant shear forces. Soleimani et al. [24] proposed a two-node axisymmetric shell element based on modified couple stress theory for the buckling analysis of cylindrical and conical shells. Tang et al. [25] developed axisymmetric finite element formulations based on Cosserat theory by introducing independent rotation degrees of freedom, establishing a pressure-dependent elastoplastic model for axisymmetric continua.

The elements constructed based on higher-order continuum theories involve second-order derivatives of displacement, thus requiring displacement shape function to at least satisfy *C*^1^ continuity in order to ensure computation convergence. Papanicolopulos et al. [26] proposed the first 8-node *C*^1^ hexahedral element based on strain gradient theory, but this element requires structured meshes and uses 24-DOF at each node, including third-order derivatives of displacement. To alleviate the interpolation difficulties caused by *C*^1^ continuity, Zhao et al. [27], based on the classical couple stress theory, combined the displacement functions of the triangular thin plate element BCIZ with the refined incompatible element ART9 to propose an 18-DOF triangular axisymmetric element that satisfies the *C*^1^ weak continuity condition. Wang et al. [28], based on the classical couple stress theory, proposed a 24-DOF quadrilateral quasi-conforming *C*^0–1^ element, but this method has only been applied to plane problems, and its effectiveness in 3D problems remains to be studied further. In addition, some researchers have employed the Lagrange multiplier method to satisfy *C*^1^ continuity in a weak form. Kwon and Lee [29] introduced rotation as an independent variable and used the Lagrange multiplier method to constrain the relationship between physical rotation and assumed rotation, thus achieving weak *C*^1^ continuity. They proposed a plane mixed element based on modified couple stress theory. Kwon and Lee [30], based on the modified couple stress theory, considered additional curvature constraints and derived weak forms that satisfy one or two constraints, applying different approximations for each independent variable domain, resulting in four 3D mixed elements that meet convergence criteria. However, the additional degrees of freedom introduced by the Lagrange multiplier method significantly increase computational costs in 3D problems and may lead to the appearance of spurious zero-energy modes in elements, thus affecting the reliability of the computational results.

This work employs the penalty function method to introduce rotational degrees of freedom as independent nodal variables, replacing the mechanical rotation determined by the displacement field with assumed rotations. By minimizing the difference between the rotation field defined by nodal displacements and the independently defined rotation field through penalty terms, the enriched isoparametric interpolation functions that satisfy *C*^0^ continuity are utilized for both the displacement and rotation fields, thereby achieving weak forms of higher-order continuity in the displacement field. For instance, Zervos [31] developed a 3D element based on micro-polar theory, using one of the parameters as a penalty parameter for analyzing strain gradient theory when specific material parameters are zero. Shang et al. [32] proposed a plane 4-node element based on modified couple stress theory using the penalty function method. Additionally, Garg and Han [33] introduced extra rotations to form a rotational gradient, presenting an axisymmetric penalty finite element method based on modified couple stress elastic theory.

Once the shape of the element mesh undergoes severe distortion, the accuracy of the finite element numerical results can drop sharply, potentially leading to issues such as shear locking and volumetric locking [34]. To address the sensitivity of mesh distortion, researchers have proposed various methods, including the hybrid/mixed FEM [35,36,37], the smoothed FEM [38,39,40], the new natural coordinate FEM [41,42,43], the unsymmetric FEM [44,45,46], the new spline FEM [47,48,49], and the hybrid-stress function FEM [50,51,52]. In addition to the usual FEM, numerous IGA (isogeometric analysis) elements have also been proposed [53,54]. These methods improve element performance to varying degrees. However, it is important to note that if the element shape becomes severely deformed, such as a convex quadrilateral degenerating into a triangle or becoming a concave quadrilateral, many models may fail. Shang et al. [32] proposed an improved unsymmetric finite element method in which the *C*^1^ continuity requirement is enforced in a weak sense using the penalty function method. The test functions for displacement and rotation are constructed using the enriched *C*^0^ isoparametric shape functions. Meanwhile, the trial functions for force-stress and couple stress are designed based on force-stress/couple stress functions that inherently satisfy the relevant governing equations. Numerical results indicate that even when the element shape is distorted into a concave quadrilateral or degenerates into a triangle, this element still exhibits good performance. Additionally, this method does not suffer from trapezoidal locking or Poisson’s ratio locking and remains invariant under coordinate rotations. Given these advantages, this work aims to apply this method to axisymmetric couple stress elasticity problems.

This work combines the consistent couple stress theory with the unsymmetric finite element method, employing a series of element techniques such as the penalty function method, analytical trial function method, and weighted residual method. A general formulation for the unsymmetric finite element method based on the consistent couple stress theory is proposed for axisymmetric problems, specifically constructing a 4-node 12-DOF axisymmetric element. Numerical examples demonstrate that this element exhibits excellent computational accuracy and convergence rate in both static and modal analysis problems, effectively simulating the size-dependent axisymmetric mechanical responses of small-scale solids.

## 2. Basic Equations of Axisymmetric Couple Stress Elasticity

**Figure 1:**
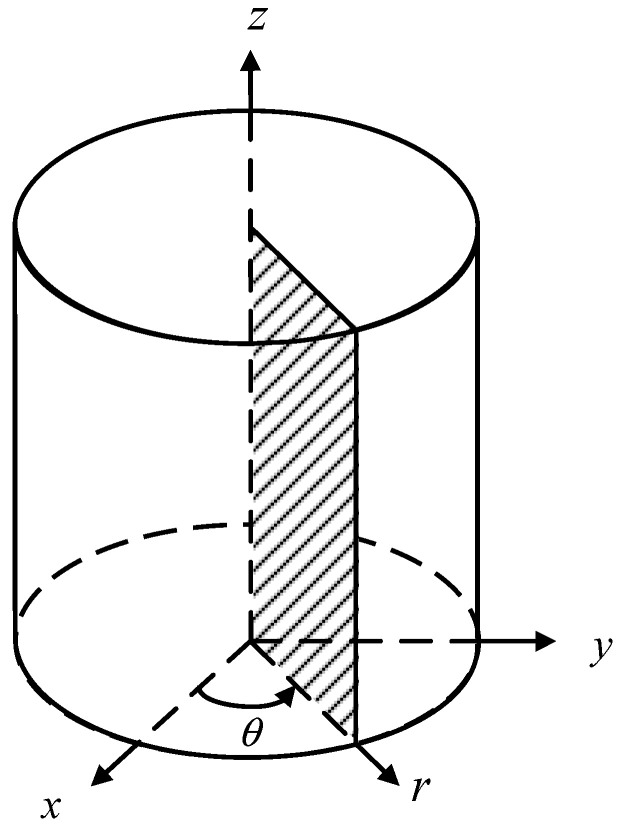
The Cartesian and cylindrical coordinate systems, with the patterned part representing an arbitrary cross-section.

Figure 1 depicts the transformation relationship between the Cartesian and cylindrical coordinate systems, as follows:

(1) $$\left\{\begin{array}{l} x=r\cos\theta \\ y=r\sin\theta \\ z=z \end{array}\right.$$

As all variables are independent of the coordinate θ, the deformation in any rz-plane represents the deformation of the entire axisymmetric body.

### 2.1. Kinematical Equations

As previously stated, this work employs the θ=0 section as the reference plane to establish the governing equations for the axisymmetric problem. In the consistent couple stress theory, when solids undergo axisymmetric deformation, the radial and axial displacements can be expressed as functions of *r* and *z*, while the circumferential displacement $u_{\theta }$ is zero:

(2) $$u_{r}=u_{r}\left(r,z\right),u_{z}=u_{z}\left(r,z\right),u_{\theta }=0$$

where the non-zero components can be rewritten in vector form as

(3) $$u=\left\{\begin{array}{c} u_{r}\\ u_{z} \end{array}\right\}=\left\{\begin{array}{c} u_{r}\left(r,z\right)\\ u_{z}\left(r,z\right) \end{array}\right\}$$

Accordingly, the only non-zero rotation component is given by

(4) $$\omega_{\theta }=\frac{1}{2}\left(\frac{\partial u_{r}}{\partial z}-\frac{\partial u_{z}}{\partial r}\right)$$

The strain is defined as the symmetric part of the displacement gradient and the non-zero components can be organized in Voigt form as

(5) $$\varepsilon =\left\{\begin{array}{c} \varepsilon_{rr}\\ \varepsilon_{\theta \theta }\\ \varepsilon_{zz}\\ 2\varepsilon_{rz} \end{array}\right\}={\left[\begin{array}{cccc} \frac{\partial u_{r}}{\partial r} & \frac{u_{r}}{r} & \frac{\partial u_{z}}{\partial z} & \frac{\partial u_{r}}{\partial z}+\frac{\partial u_{z}}{\partial r} \end{array}\right]}^{T}$$

The micro curvature is defined as the skew-symmetric part of the displacement gradient and the non-zero components can be organized in Voigt form as

(6) $$\kappa =\left\{\begin{array}{c} -2\kappa_{\theta z}\\ -2\kappa_{r\theta } \end{array}\right\}=\left\{\begin{array}{c} -\frac{\partial \omega_{\theta }}{\partial z}\\ \frac{\partial \omega_{\theta }}{\partial r}+\frac{\omega_{\theta }}{r} \end{array}\right\}$$

### 2.2. Constitutive Equations

In the CCST [12], the force-stress tensor is non-symmetric and in general can be divided into the symmetric part and the skew-symmetric part:

(7) $$\sigma =\sigma_{\mathrm{symm}}+\sigma_{\mathrm{skew}}$$

with

(8) $$\sigma_{\mathrm{symm}}={\left[\begin{array}{cccc} \sigma_{\left(rr\right)} & \sigma_{\left(\theta \theta \right)} & \sigma_{\left(zz\right)} & \sigma_{\left(rz\right)} \end{array}\right]}^{T}$$

(9) $$\sigma_{skew}={\left[\begin{array}{cccc} 0 & 0 & 0 & \sigma_{\left[rz\right]} \end{array}\right]}^{T}$$

It is important to note that only the symmetric part of the force-stress and the couple stress contribute to the strain energy, while the skew-symmetric part of the force-stress does not generate strain energy. The symmetric part of the force-stress is obtained from the strain using the constitutive equation:

(10) $$\sigma_{\mathrm{symm}}=C_{n}\varepsilon$$

And the couple stress can be derived from the curvature using the following relationship:

(11) $$m=\left\{\begin{array}{c} m_{\theta z}\\ m_{r\theta } \end{array}\right\}=C_{c}\kappa$$

For isotropic materials, $C_{n}$ and $C_{c}$ respectively read

(12) $$C_{n}=\frac{E}{\left(1-2v\right)\left(1+v\right)}\left[\begin{array}{cccc} 1-v & v & v & 0\\ v & 1-v & v & 0\\ v & v & 1-v & 0\\ 0 & 0 & 0 & \frac{1-2v}{2} \end{array}\right]$$

(13) $$C_{c}=\left[\begin{array}{cc} 4Gl^{2} & 0\\ 0 & 4Gl^{2} \end{array}\right]$$

where E is Young’s modulus, v is Poisson’s ratio, G is the shear modulus and l is additional material length scale parameter for representing the size dependence.

### 2.3. Equilibrium Equations

In the CCST, the body couple can be transformed into an equivalent body force and surface force on the boundary [12]. Thus, the equilibrium equations that the symmetric part of the force-stress and couple stress should satisfy do not include the body couple terms:

(14) $$\left\{\begin{array}{c} \frac{\partial \sigma_{\left(rr\right)}}{\partial r}+\frac{\partial \sigma_{\left(zr\right)}}{\partial z}+\frac{\partial \sigma_{\left[zr\right]}}{\partial z}+\frac{\sigma_{\left(rr\right)}-\sigma_{\left(\theta \theta \right)}}{r}+f_{r}=0\\ \frac{\partial \sigma_{\left(rz\right)}}{\partial r}+\frac{\partial \sigma_{\left[rz\right]}}{\partial r}+\frac{\partial \sigma_{\left(zz\right)}}{\partial z}+\frac{\sigma_{\left(rz\right)}}{r}+\frac{\sigma_{\left[rz\right]}}{r}+f_{z}=0 \end{array}\right.$$

(15) $$\sigma_{\left[rz\right]}-\frac{1}{2}\frac{\partial m_{r\theta }}{\partial r}+\frac{1}{2}\frac{\partial m_{\theta z}}{\partial z}=0$$

in which $f_{r}$ and $f_{z}$ are the external body loads. Then, by substituting Equation (15) into Equation (14), we can further obtain

(16) $$\left\{\begin{array}{l} \begin{array}{l} \frac{\partial \sigma_{\left(rr\right)}}{\partial r}+\frac{\partial \sigma_{\left(rz\right)}}{\partial z}+\frac{\sigma_{\left(rr\right)}-\sigma_{\left(\theta \theta \right)}}{r}+\frac{1}{2}\frac{\partial^{2}m_{\theta z}}{\partial z^{2}}-\frac{1}{2}\frac{\partial^{2}m_{r\theta }}{\partial r\partial z}+f_{r}=0 \end{array}\\ \begin{array}{l} \frac{\partial \sigma_{\left(rz\right)}}{\partial r}+\frac{\partial \sigma_{\left(zz\right)}}{\partial z}+\frac{\sigma_{\left(rz\right)}}{r}-\frac{1}{2}\frac{\partial^{2}m_{\theta z}}{\partial r\partial z}+\frac{1}{2}\frac{\partial^{2}m_{r\theta }}{\partial r^{2}}-\frac{1}{2}\frac{\partial m_{\theta z}}{r\partial z}+\frac{1}{2}\frac{\partial m_{r\theta }}{r\partial r}+f_{z}=0 \end{array} \end{array}\right.$$

### 2.4. Boundary Conditions

In the CCST, the force boundary conditions are

(17) $$\left\{\begin{array}{c} \sigma_{rr}n_{r}+\sigma_{rz}n_{z}={\bar{t}}_{r}\\ \sigma_{rz}n_{r}+\sigma_{zz}n_{z}={\bar{t}}_{z}\\ m_{r\theta }n_{r}-m_{\theta z}n_{z}={\bar{M}}_{\theta } \end{array}\right.$$

where ${\bar{t}}_{r}$ and ${\bar{t}}_{z}$ represent the surface forces applied on the boundary surface, and ${\bar{M}}_{\theta }$ denotes the couple moments applied on the boundary surface. $n_{r}$ and $n_{z}$ are the direction cosines of the boundary normal with respect to the *r*-axis and *z*-axis, respectively.

The displacement boundary conditions are

(18) $$\left\{\begin{array}{c} u_{r}={\bar{u}}_{r}\\ u_{z}={\bar{u}}_{z}\\ \omega_{\theta }={\bar{\omega }}_{\theta } \end{array}\right.$$

where ${\bar{u}}_{r}$ and ${\bar{u}}_{z}$ are the given boundary displacements in *r* and *z* directions separately, and ${\bar{\omega }}_{\theta }$ is the given boundary rotation.

### 2.5. The Virtual Work Principle

The principle of virtual work with respect to the cylindrical coordinate system for axisymmetric problems based on the consistent couple stress theory can be expressed as follows:

(19) $$\delta \Pi =\delta \Pi_{\mathrm{kin}}+\delta \Pi_{\mathrm{in}}-\delta \Pi_{\mathrm{out}}=0$$

with

(20) $$\delta \Pi_{\mathrm{kin}}=2\pi \iint_{A}\delta u^{T}\ddot{u}\rho rdA$$

(21) $$\delta \Pi_{\mathrm{in}}=2\pi \iint_{A}\delta \varepsilon {\left(u\right)}^{T}\sigma_{\mathrm{symm}}rdA+2\pi \iint_{A}\delta \kappa {\left(u\right)}^{T}mrdA$$

(22) $$\delta \Pi_{\mathrm{out}}=2\pi \iint_{A}\delta u^{T}frdA+2\pi \int_{\Gamma }\delta u^{T}\bar{t}rd\Gamma +2\pi \int_{\Gamma }\delta \omega^{T}\bar{M}rd\Gamma$$

where A denotes the representative section plane bounded by the edge Γ, as depicted in Figure 1, and ρ represents the density.

## 3. Finite Element Formulation

### 3.1. The Modified Virtual Work Principle

In the CCST, the curvature is the second derivative of displacement. Therefore, the test function for displacement should at least satisfy *C*^1^ continuity. This leads to two challenges: the first is the generation of a large number of DOFs, which increases computational costs; the second is the numerical complexity associated with solving fourth-order differential equations. To overcome these challenges, the penalty function method is employed, introducing rotational degrees of freedom as independent nodal variables. This approach implies that the rotation vector determined by the displacement field will be replaced by the assumed rotation in the finite element formulation. By using the penalty term, the difference between the rotation field defined by nodal displacement and the independently defined rotation field is minimized, thereby achieving weak forms of high-order continuity for the displacement field. The penalty term is given as follows:

(23) $$\delta \Pi_{\mathrm{penalty}}=2\pi k\iint_{A}\delta \Lambda^{T}\Lambda rdA$$

where k is the penalty parameter and Λ takes the form

(24) $$\Lambda =\varphi_{\theta }-\omega_{\theta }\left(u\right)$$

where $\varphi_{\theta }$ is the rotation field interpolated by the element nodal rotation DOFs. Thus, the virtual work principle shown in Equation (19) can be updated as follows:

(25) $$\delta \Pi^{*}=\delta \Pi_{\mathrm{kin}}^{\prime }+\delta \Pi_{\mathrm{in}}^{\prime }-\delta \Pi_{\mathrm{out}}^{\prime }+\delta \Pi_{\mathrm{penalty}}=0$$

in that $\delta \Pi_{\mathrm{kin}}^{\prime }$, $\delta \Pi_{\mathrm{in}}^{\prime }$ and $\delta \Pi_{\mathrm{out}}^{\prime }$ are given by

(26) $$\delta \Pi_{\mathrm{kin}}^{\prime }=2\pi \iint_{A}\delta u^{T}\ddot{u}\rho rdA$$

(27) $$\delta \Pi_{\mathrm{in}}^{\prime }=2\pi \iint_{A}\delta \varepsilon {\left(u\right)}^{T}\sigma_{\mathrm{symm}}rdA+2\pi \iint_{A}\delta \kappa {\left(\varphi_{\theta }\right)}^{T}mrdA$$

(28) $$\delta \Pi_{\mathrm{out}}^{\prime }=2\pi \iint_{A}\delta u^{T}frdA+2\pi \int_{\Gamma }\delta u^{T}\bar{t}rd\Gamma +2\pi \int_{\Gamma }\delta {\varphi_{\theta }}^{T}\bar{M}rd\Gamma$$

### 3.2. The New 4-Node 12-DOF Axisymmetric Element

**Figure 2:**
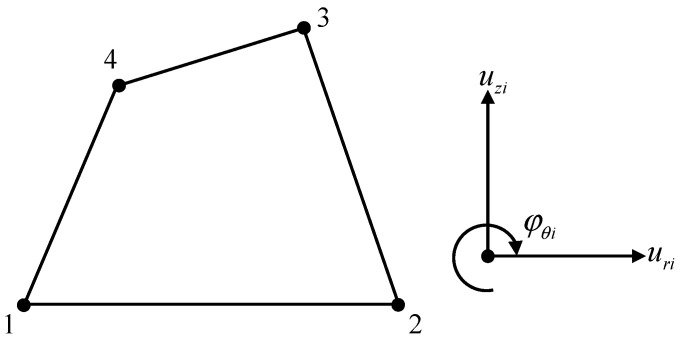
The 4-node 12-DOF axisymmetric element.

As shown in Figure 2, for axisymmetric problems based on the consistent couple stress theory, the 4-node 12-DOF axisymmetric element is constructed, where each node has two displacement DOFs and one rotation DOF. The nodal DOF vector for the element can be expressed as

(29) $$q^{e}={\left[\begin{array}{cccc} q_{1}^{e} & q_{2}^{e} & q_{3}^{e} & q_{4}^{e} \end{array}\right]}^{T}$$

with

(30) $$q_{i}^{e}=\left[\begin{array}{ccc} u_{ri} & u_{zi} & \varphi_{\theta i} \end{array}\right],\;\;\;i=1\sim 4$$

As previously mentioned, the penalty function method is employed in the general formulation of finite element construction to satisfy the requirement of *C*^1^ continuity for displacements in a weak form. The standard isoparametric interpolation functions serve as a feasible choice, and further enhancement of the displacement interpolation order is achieved through the introduction of link interpolation techniques [55]. The displacement test functions are constructed as follows:

(31) $$u=Nq^{e},\;\;\;N=\left[\begin{array}{cccc} N_{1} & N_{2} & N_{3} & N_{4} \end{array}\right]$$

with

(32) $$N_{i}=\left[\begin{array}{ccc} N_{i} & 0 & \frac{1}{2}N_{i}\left(z-z_{i}\right)\\ 0 & N_{i} & -\frac{1}{2}N_{i}\left(r-r_{i}\right) \end{array}\right]\;,\;\;\;i=1\sim 4$$

where $\left(r_{i},z_{i}\right)$ are the cylindrical coordinates of node i and

(33) $$r=\sum_{i=1}^{4}N_{i}r_{i},\;z=\sum_{i=1}^{4}N_{i}z_{i}$$

in which $N_{i}$ $\left(i=1\sim 4\right)$ are the shape functions of the standard 4-node isoparametric element:

(34) $$\left\{\begin{array}{c} N_{1}=\frac{1}{4}(1-\xi )(1-\eta )\\ N_{2}=\frac{1}{4}(1+\xi )(1-\eta )\\ N_{3}=\frac{1}{4}(1+\xi )(1+\eta )\\ N_{4}=\frac{1}{4}(1-\xi )(1+\eta ) \end{array}\right.$$

in which $\left(\xi ,\eta \right)$ are the isoparametric coordinates. By substituting the assumed displacement field into Equation (5), the corresponding strain can be obtained:

(35) $$\varepsilon =B^{n}q^{e},\;\;\;B^{n}=\left[\begin{array}{c} \begin{array}{cccc} B_{1}^{n} & B_{2}^{n} & B_{3}^{n} & B_{4}^{n} \end{array} \end{array}\right]$$

in which

(36) $$B_{i}^{n}=\left[\begin{array}{ccc} N_{i,r} & 0 & \frac{1}{2}N_{i,r}\left(z-z_{i}\right)\\ \frac{N_{i}}{r} & 0 & \frac{1}{2}\frac{N_{i}\left(z-z_{i}\right)}{r}\\ 0 & N_{i,z} & -\frac{1}{2}N_{i,z}\left(r-r_{i}\right)\\ N_{i,z} & N_{i,r} & \frac{1}{2}N_{i,z}\left(z-z_{i}\right)-\frac{1}{2}N_{i,r}\left(r-r_{i}\right) \end{array}\right],\;i=1∼4$$

In addition, the independently introduced rotation $\varphi_{\theta }$ is also formulated using the *C*^0^ isoparametric shape function:

(37) $$\varphi_{\theta }=N^{\varphi }q^{e},\;\;\;N^{\varphi }=\left[\begin{array}{cccc} N_{1}^{\varphi } & N_{2}^{\varphi } & N_{3}^{\varphi } & N_{4}^{\varphi } \end{array}\right]$$

with

(38) $$N_{i}^{\varphi }=\left[\begin{array}{ccc} 0 & 0 & N_{i} \end{array}\right],\;\;\;i=1\sim 4$$

By substituting the assumed displacement field into Equation (6), the curvature is given by

(39) $$\kappa =B^{c}q^{e},\;\;\;B^{c}=\left[\begin{array}{c} \begin{array}{cccc} B_{1}^{c} & B_{2}^{c} & B_{3}^{c} & B_{4}^{c} \end{array} \end{array}\right]$$

with

(40) $$B_{i}^{c}=\left[\begin{array}{ccc} 0 & 0 & -N_{i,z}\\ 0 & 0 & N_{i,r}+\frac{N_{i}}{r} \end{array}\right],\;\;\;i=1∼4$$

The acceleration field in the dynamic formulation is constructed using the same interpolation method as that of the displacement field:

(41) $$\ddot{u}=\left\{\begin{array}{c} {\ddot{u}}_{r}\\ {\ddot{u}}_{z} \end{array}\right\}=N{\ddot{q}}^{e}$$

Additionally, the specific formulation for the penalty function is given as follows:

(42) $$\Lambda =N^{\Lambda }q^{e},\;\;\;N^{\Lambda }=\left[\begin{array}{cccc} N_{1}^{\Lambda } & N_{2}^{\Lambda } & N_{3}^{\Lambda } & N_{4}^{\Lambda } \end{array}\right]$$

with

(43) $$B_{i}^{\Lambda }=\frac{1}{2}\left[\begin{array}{ccc} -N_{i,z} & N_{i,r} & -\frac{1}{2}N_{i,z}\left(z-z_{i}\right)-\frac{1}{2}N_{i,r}\left(r-r_{i}\right)+N_{i} \end{array}\right],\;\;\;\;i=1∼4$$

Once the shape of the element mesh becomes distorted, the accuracy of the finite element numerical results can deteriorate sharply, potentially leading to issues such as shear locking and volume locking. To address the sensitivity of mesh distortion, the unsymmetric finite element method is employed, in which the test functions for the displacement field and the trial functions for the force-stress are distinct. The test function for displacement is constructed based on local isoparametric coordinates, while the trial function for force-stress is formulated using the force-stress field that a priori satisfies the equilibrium equations.

Considering that external forces are represented as equivalent nodal forces in the finite element method, the body force terms can be omitted when deriving the force-stress trial function that satisfies the equilibrium differential equations to simplify the solution process. Although this approach introduces some error during the numerical computation of the element, this error can be gradually reduced as the mesh is refined. After neglecting the body force terms, the equilibrium equations can be divided into two parts:

(44) $$\left\{\begin{array}{l} \frac{\partial \sigma_{\left(rr\right)}}{\partial r}+\frac{\partial \sigma_{\left(rz\right)}}{\partial z}+\frac{\sigma_{\left(rr\right)}-\sigma_{\left(\theta \theta \right)}}{r}=0\\ \frac{\partial \sigma_{\left(rz\right)}}{\partial r}+\frac{\partial \sigma_{\left(zz\right)}}{\partial z}+\frac{\sigma_{\left(rz\right)}}{r}=0 \end{array}\right.$$

(45) $$\left\{\begin{array}{l} \frac{1}{2}\frac{\partial^{2}m_{\theta z}}{\partial z^{2}}-\frac{1}{2}\frac{\partial^{2}m_{r\theta }}{\partial r\partial z}=0\\ -\frac{1}{2}\frac{\partial^{2}m_{\theta z}}{\partial r\partial z}+\frac{1}{2}\frac{\partial^{2}m_{r\theta }}{\partial r^{2}}-\frac{1}{2}\frac{\partial m_{\theta z}}{r\partial z}+\frac{1}{2}\frac{\partial m_{r\theta }}{r\partial r}=0 \end{array}\right.$$

It is observed that for the current quadrilateral 4-node element used for axisymmetric problems, the couple stress field designed based on functions that satisfy the relevant equations does not significantly enhance the element’s performance. Therefore, to ensure efficiency and conciseness, while only Equation (44) will be solved, the interpolation functions for couple stress are derived from the constitutive relations:

(46) $$m=S^{c}q^{e},\;\;\;S^{c}=C_{c}B^{c}$$

Since Equation (44) is a linear differential equation with a coefficient of 1/r, the construction of the force-stress field can be complex and may not yield optimal results. Therefore, multiplying the equations in Equation (44) by r provides a new form of the equilibrium equation:

(47) $$\left\{\begin{array}{l} \frac{\partial \left(r\sigma_{\left(rr\right)}\right)}{\partial r}+\frac{\partial \left(r\sigma_{\left(rz\right)}\right)}{\partial z}-\sigma_{\left(\theta \theta \right)}=0\\ \frac{\partial \left(r\sigma_{\left(rz\right)}\right)}{\partial r}+\frac{\partial \left(r\sigma_{\left(zz\right)}\right)}{\partial z}=0 \end{array}\right.$$

Then, the items $r\sigma_{\left(rr\right)}$, $\sigma_{\left(\theta \theta \right)}$, $r\sigma_{\left(zz\right)}$ and $r\sigma_{\left(rz\right)}$ are assumed as the following polynomials, in which twenty-one coefficients are introduced:

(48) $$\left\{\begin{array}{l} r\sigma_{\left(rr\right)}=a_{1}+a_{2}r+a_{3}z+a_{4}r^{2}+a_{5}z^{2}+a_{6}rz\\ \sigma_{\left(\theta \theta \right)}=b_{1}+b_{2}r+b_{3}z\\ r\sigma_{\left(zz\right)}=c_{1}+c_{2}r+c_{3}z+c_{4}r^{2}+c_{5}z^{2}+c_{6}rz\\ r\sigma_{\left(rz\right)}=d_{1}+d_{2}r+d_{3}z+d_{4}r^{2}+d_{5}z^{2}+d_{6}rz \end{array}\right.$$

Substituting Equation (48) into Equation (47) yields:

(49) $$b_{1}=a_{2}+d_{3},\;\;b_{2}=2a_{4}+d_{6},\;\;b_{3}=a_{6}+2d_{5},\;\;d_{2}=-c_{3},\;\;d_{4}=-\frac{1}{2}c_{6},\;\;d_{6}=-2c_{5}$$

from which we can obtain

(50) $$\left\{\begin{array}{l} \sigma_{\left(rr\right)}=a_{1}\frac{1}{r}+a_{2}+a_{3}\frac{z}{r}+a_{4}r+a_{5}\frac{z^{2}}{r}+a_{6}z\\ \sigma_{\left(\theta \theta \right)}=\left(a_{2}+d_{3}\right)+\left(2a_{4}-2c_{5}\right)r+\left(a_{6}+2d_{5}\right)z\\ \sigma_{\left(zz\right)}=c_{1}\frac{1}{r}+c_{2}+c_{3}\frac{z}{r}+c_{4}r+c_{5}\frac{z^{2}}{r}+c_{6}z\\ \sigma_{\left(rz\right)}=d_{1}\frac{1}{r}-c_{3}+d_{3}\frac{z}{r}-\frac{1}{2}c_{6}r+d_{5}\frac{z^{2}}{r}-2c_{5}z \end{array}\right.$$

By combining Equation (50), the fifteen terms of force-stress solutions are obtained, as summarized in Table 1, for designing the element’s force-stress trial function.

To ensure the element has the correct rank and enhance computational efficiency, the first ten terms of the force-stress solutions are selected to construct the force-stress trial function for the axisymmetric element, in accordance with the method outlined in reference [56]. The element’s force-stress trial function is expressed as

(51) $$\sigma_{\mathrm{symm}}=\left\{\begin{array}{c} \sigma_{\left(rr\right)}\\ \sigma_{\left(\theta \theta \right)}\\ \sigma_{\left(zz\right)}\\ \sigma_{\left(rz\right)} \end{array}\right\}=H\alpha ,\;H=\left[\begin{array}{cccc} H_{1} & H_{2} & \cdots & H_{10} \end{array}\right]$$

with

(52) $$\alpha ={\left[\begin{array}{cccc} \alpha_{1} & \alpha_{2} & \ldots & \alpha_{10} \end{array}\right]}^{T}$$

The relationship between the undetermined coefficients of the assumed force-stress field and the element nodal degrees of freedom is established using the weighted residual method:

(53) $$2\pi \iint_{A}W\left(\hat{\varepsilon }-\bar{\varepsilon }\right)rdA=0$$

in which W is weighted test function. The physical significance is that when the force-stress field is specified, the work performedby the two different strain fields, $\hat{\varepsilon }$ and $\bar{\varepsilon }$, is equal. Here, the weighted test function W is set as H. $\bar{\varepsilon }$ is the strain produced by the assumed force-stress field $\sigma_{\mathrm{symm}}$:

(54) $$\bar{\varepsilon }=C_{n}^{-1}\sigma_{\mathrm{symm}}=C_{n}^{-1}H\alpha$$

And $\hat{\varepsilon }$ is determined as the strain related to the enriched isoparametric-based displacement field:

(55) $$\hat{\varepsilon }=\varepsilon =B^{n}q^{e}$$

From the above relationship, it can be derived that:

(56) $$\alpha =M^{-1}Vq^{e}$$

with

(57) $$V=\iint_{A}H^{T}B^{n}rdA,\;\;\;M=\iint_{A}H^{T}C_{n}^{-1}HrdA$$

Substituting Equation (56) into Equation (51) yields the force-stress trial function:

(58) $$\sigma_{\mathrm{symm}}=S^{n}q^{e},\;\;\;S^{n}=HM^{-1}V$$

Finally, by making use of the foregoing definitions into the modified virtual work principle in Equation (25), the element mass matrix, the element stiffness matrix and the element equivalent load vector are expressed, respectively, as

(59) $$M^{e}=2\pi \iint_{A}N^{T}N\rho rdA$$

(60) $$K^{e}=2\pi \left(\iint_{A}B^{nT}S^{n}rdA+\iint_{A}B^{cT}S^{c}rdA+k\iint_{A}N^{\Lambda T}N^{\Lambda }rdA\right)$$

(61) $$P^{e}=2\pi \left(\iint_{A}N^{T}frdA+\int_{\Gamma }N^{T}\bar{t}rd\Gamma +\int_{\Gamma }N^{\varphi T}\bar{M}rd\Gamma \right)$$

The rotation determined by the displacement field and the rotation obtained directly through nodal variable interpolation have different interpolation orders. In elements where the shape functions are linear, using full integration for the penalty stiffness term may lead to locking issues. To address this problem, the single point integration scheme is employed for the penalty stiffness term. Except for the last penalty term in Equation (60), the other terms in Equations (59), (60) and (61)—are calculated using the 3 × 3 Gauss integral scheme. In addition, according to the results of a parameter analysis, the penalty parameter k is set as $10^{5}G$.

## 4. Numerical Tests

Several numerical tests are examined to assess the performance of the new element in the simulation of the size-dependent axisymmetric deformation of small-scale solids. When the analytical solution is not available for some examples, the numerical results obtained by using the 20-node hexahedral element [57] are provided as the reference solutions.

### 4.1. The Circular Plate Under Uniformly Distributed Pressure

**Figure 3:**
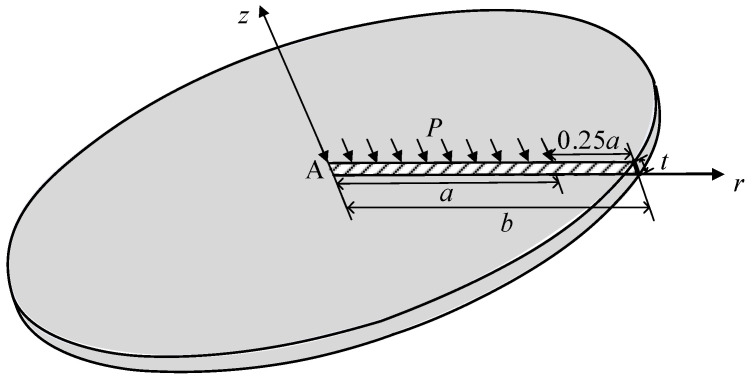
The model of the circular plate under uniformly distributed pressure.

As illustrated in Figure 3, this work considers the deformation of the circular plate with a radius of *b* = 125 μm and thickness *t.* For 0 ≤ *r* ≤ *a*, the upper surface of the plate is subjected to a uniform pressure of *P* = 1 N/mm^2^, and for *a* < *r* ≤ *b*, the degrees of freedom on the lower surface are fully constrained, with all other surfaces being free. In this analysis, all numerical simulations are performed with an aspect ratio of *a*/*t* = 10. The elastic modulus of the material is given by *E* = 1.5 GPa, while Poisson’s ratio is *ν* = 0.355. The circular plate is discretized using an *M* × *N* mesh, where *M* denotes the number of elements in the longitudinal direction and *N* denotes the number of elements in the radial direction. The specific meshing schemes employed are 2 × 20, 4 × 40, 8 × 80 and 16 × 160.

Firstly, this work investigates the convergence of the deflection $u_{zA}$ at point A (0.5), situated at the center of the left end of a circular plate with a thickness of *t* = 10 μm, subjected to a uniformly distributed load. In the absence of an exact analytical solution, the numerical solution obtained using the 95,000 hexahedral elements based on the consistent couple stress theory, as proposed by Wu [57], is adopted as the reference solution. As illustrated in Table 2, an increase in the number of mesh elements results in a progressive convergence of the deflection at point A towards the reference solution. Furthermore, as the material length scale *l* increases, the deflection at point A decreases, thereby demonstrating the influence of the size effect.

**Figure 4:**
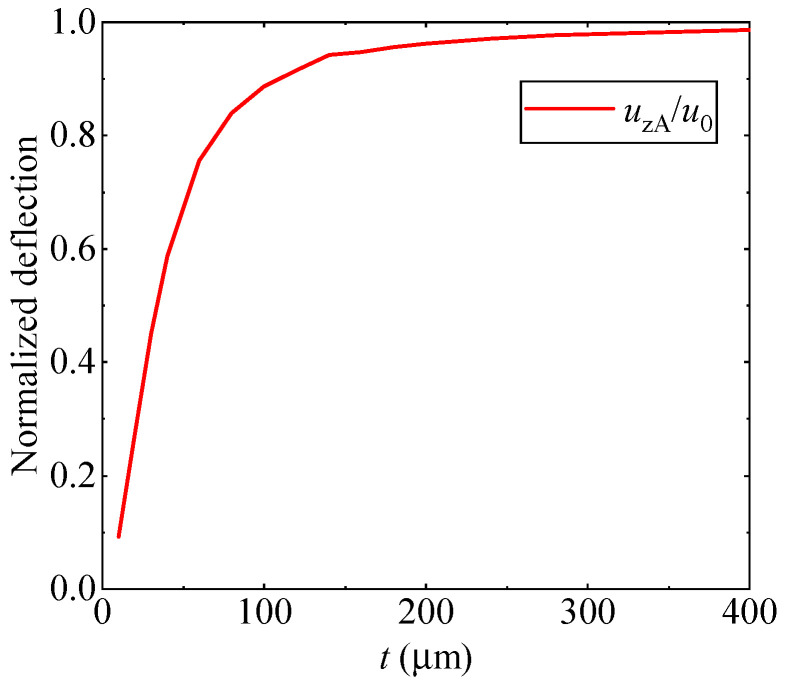
The variation in normalized deflection with plate thickness.

As illustrated in Table 2, the use of 16 elements along the thickness direction and 160 elements along the radial direction produces sufficiently accurate results. Consequently, the 16 × 160 mesh is employed to calculate the deflection at point A based on the consistent couple stress theory, with the deflection normalized by that obtained from classical continuum theory $u_{0}$. In this analysis, the material length scale parameter is *l* = 8.35 μm, and the plate thickness ranges from a few micrometers to several hundred micrometers. Figure 4 clearly demonstrates that as the plate thickness increases from 10 μm to 400 μm, the deflection ratio rises significantly, while the stiffness decreases by nearly an order of magnitude. As the thickness increases further, the normalized deflection approaches unity, suggesting that size effects become negligible for thicker plates and that the results align closely with classical continuum theory.

### 4.2. Axisymmetric Shear Deformation of the Hollow Circular Plate

**Figure 5:**
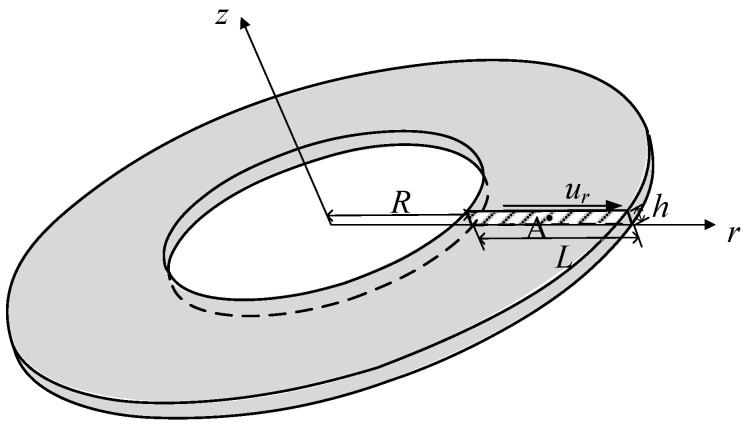
The hollow circular plate.

As shown in Figure 5, the hollow circular plate is clamped at the bottom surface while the boundary condition at its top surface is set as

(62) $$u_{r}=1\;\mu m,\;\;\;u_{z}=\omega_{\theta }=0$$

**Figure 6:**
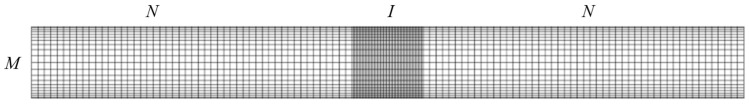
The typical mesh of the hollow circular plate.

The geometrical parameters are *R* = 1.0 mm, *L* = 1.0 mm and *h* = 0.1 mm. Considering composite materials, for *R* ≤ *r* ≤ *R* + *L*/2, the Young’s modulus and Poisson’s ratio are *E* = 1.44 GPa and *ν* = 0.38, and for *R* + *L*/2 < *r* ≤ *R* + *L*, the Young’s modulus and Poisson’s ratio are *E* = 70 GPa and *ν* = 0.3. As shown in Figure 6, the representative cross section is modeled by the new element with the mesh *M* × *N* × *I*. Due to the potential for abrupt changes at the interface between the two materials, mesh refinement is applied near the connection area, in which the range of *I* is for 1.45 mm ≤ *r* ≤ 1.55 mm. Moreover, considering the large rotation gradient near the top and bottom surfaces, the bias mesh is used for mesh generation, with denser meshing applied near the top and bottom surfaces.

**Figure 7:**
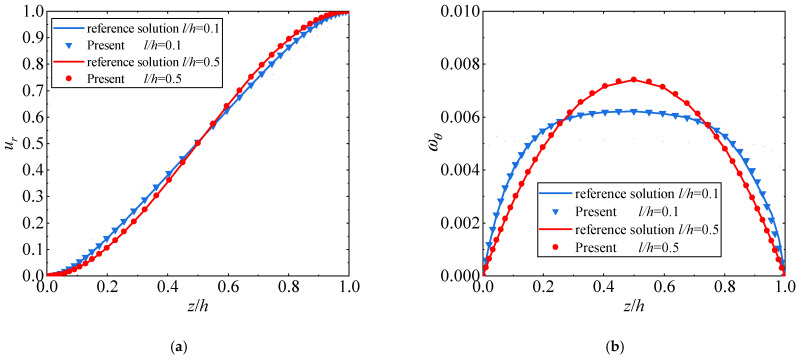
(**a**) The distributions of the displacement of the hollow circular plate; (**b**) the distributions of the rotation of the hollow circular plate.

Since no analytical solution is available for this case, the numerical solution based on the consistent couple stress theory using 72,000 hexahedral elements is employed as a reference solution [57]. First, the convergence of the new element is checked and the numerical results calculated the characteristic point A $\left(r=R+L/2,\;z=h/2\right)$ are summarized in Table 3. It can be seen that the results of the displacement and rotation converge into the reference solutions very rapidly. Second, in accordance with the convergence test, the mesh composed of 40 × 50 × 50 elements is used to predict the distributions of the displacement and rotation along the vertical path *r* = 1.5 mm. As evident in Figure 7, the results of the new element are in good agreement with the reference solutions, capturing the size dependence well.

### 4.3. The Circular Cylinder with a Spherical Hole Under Tensile Load

**Figure 8:**
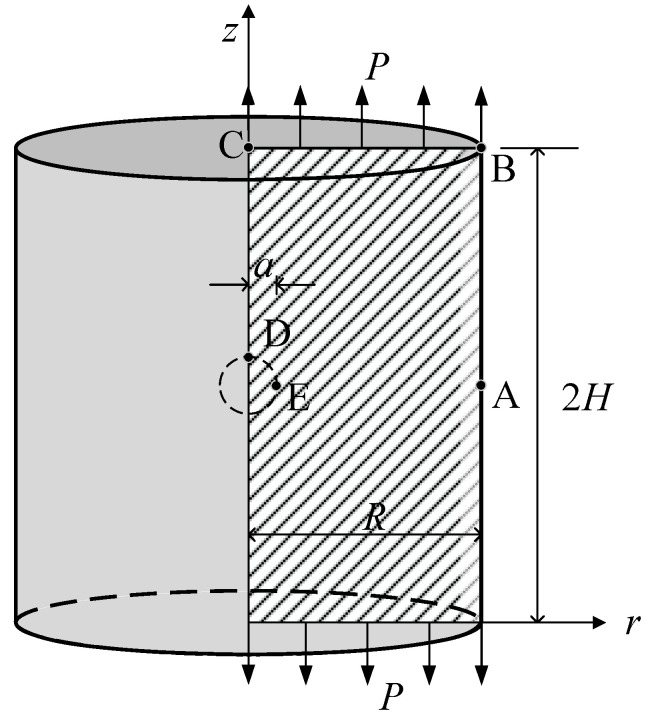
The model of the circular cylinder with a spherical hole.

**Figure 9:**
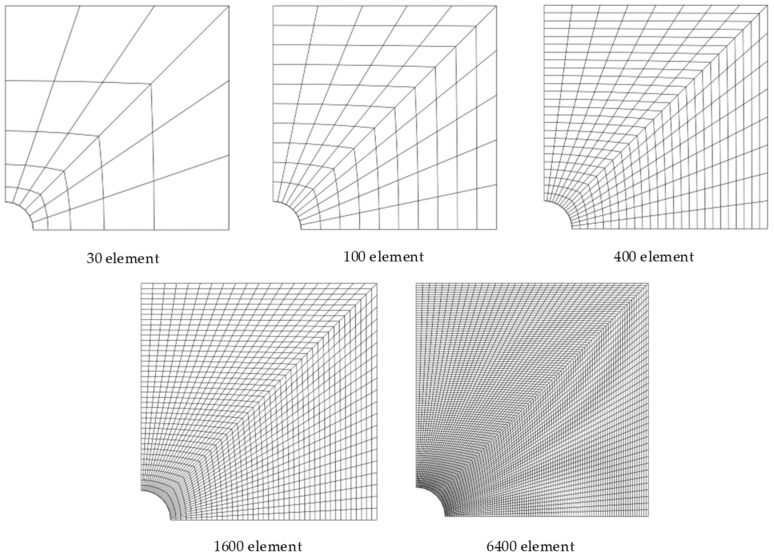
The bias meshes used for the circular cylinder with a spherical hole under tensile load.

As shown in Figure 8, when a cylindrical body with a central spherical hole is subjected to uniform tensile loading, stress concentration occurs near the spherical hole. The cylinder has a radius *R* = 8 μm, an internal spherical hole radius *a* =1 μm, and a height of 2 *H* = 16 μm. Due to symmetry, only half of the structure is considered. A uniform tensile load *P* = 1 μN/μm^2^ is applied at the top and bottom, while symmetric boundary conditions are imposed on edges AE and CD, with the remaining surfaces being free boundaries. The elastic modulus *E* = 1.5 GPa and Poisson’s ratio *ν* = 0.355. A bias mesh is used, with fine discretization around the spherical hole and coarse discretization farther away. Figure 9 shows the mesh division schematic for the half-structure model, with specific mesh division schemes consisting of 30 elements, 100 elements, 400 elements, 1600 elements, and 6400 elements, respectively.

First, the effect of the material length scale parameter *l* on the displacement at point E and the displacement at point D is investigated. Since no analytical solution is available for this case, the numerical solution based on the consistent couple stress theory using 92,700 hexahedral elements is employed as a reference solution [57]. As shown in Table 4, the displacements at points E and D gradually converge to the reference solution as the number of mesh elements increases. Additionally, as *l*/*r* increases, the displacements at points E and D decrease, demonstrating a clear size effect.

**Figure 10:**
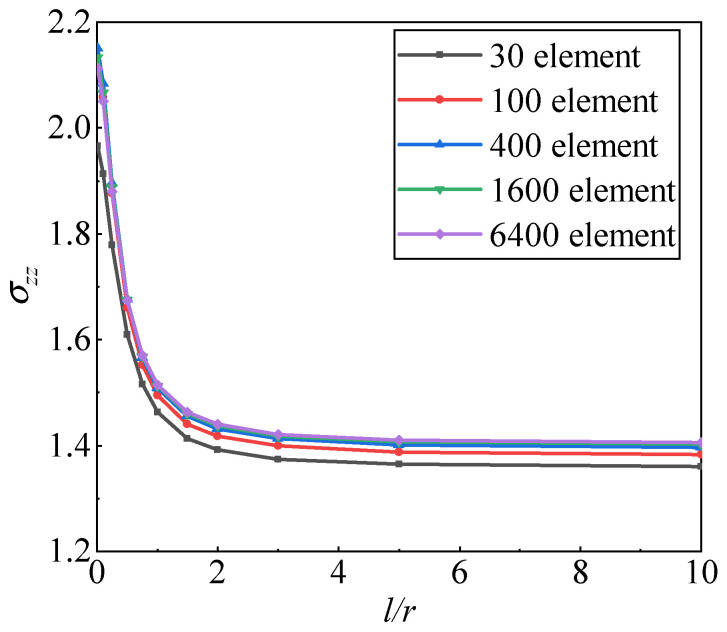
The convergence results of the normal force-stress *σ*_zz_ (MPa) at point E with respect to *l*/*r*.

Next, the effect of the material length scale parameter on the force-stress $\sigma_{\mathrm{zz}}$ at point E is investigated. As shown in Figure 10, the force-stress $\sigma_{\mathrm{zz}}$ at point E converges rapidly as the mesh is refined. Moreover, as the material length scale parameter *l* increases, the force-stress $\sigma_{\mathrm{zz}}$ at point E gradually decreases. These results indicate that with the increase in *l* /*r*, the influence of couple stress and mean curvature on the strain energy becomes more significant, while the influence of force-stress and strain on the strain energy diminishes. The variation in force-stress $\sigma_{\mathrm{zz}}$ at point E suggests that when the radius of the spherical hole approaches the material length scale parameter *l*, a pronounced size effect occurs.

### 4.4. The Modal Analysis of the Circular Plate

**Figure 11:**
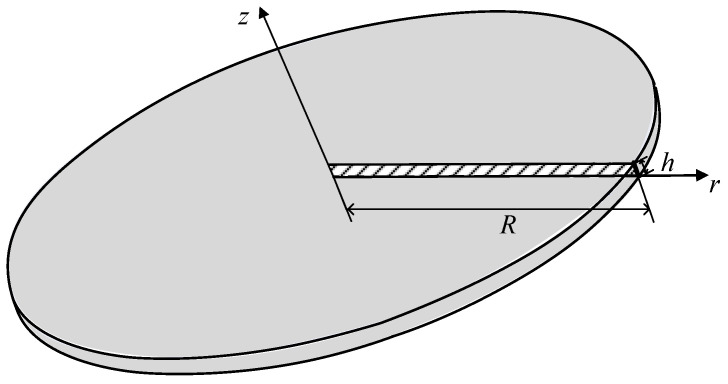
The model of the circular plate.

As shown in Figure 11, the modal analysis of a simply supported circular plate and a clamped circular plate is performed. The radius of the plate is *R* = 5 mm and the thickness is *h* = 0.1 mm. The material properties are as follows: elastic modulus *E* = 1.44 GPa, Poisson’s ratio *ν* = 0.38, and density *ρ* = 1220 kg/m^3^. The boundary condition for the simply supported edge of the circular plate is

(63) $$u_{z}=0\;\mathrm{at}\;\mathrm{the}\;\mathrm{surface}\;r=5\mathrm{mm}$$

and the boundary conditions for the clamped edge of the circular plate are

(64) $$u_{r}=u_{z}=\omega_{\theta }=0\;\mathrm{at}\;\mathrm{the}\;\mathrm{surface}\;r=5\mathrm{mm}$$

The geometry is discretized by *M* × *N* elements, where *M* is the number of elements along the thickness and *N* is the number of elements along the radial direction. The mesh division schemes of 1 × 20, 2 × 100, 5 × 100, 8 × 400, and 10 × 500 correspond to 20, 200, 1000, 3200, and 5000 elements, respectively.

Since no analytical solution is available for this case, the numerical results from Mao et al. [58] are used as reference solutions. The natural frequency ϑ is nondimensionalized as follows:

(65) $$\bar{\vartheta }=\vartheta R^{2}\sqrt{\frac{\rho h}{D}}$$

where *D* is the bending stiffness of the circular plate, and $\bar{\vartheta }$ is the nondimensionalized natural frequency of the circular plate. Table 5 and Table 6 present the convergence results of the first three nondimensional natural frequencies for simply supported and clamped circular plates, respectively. It can be seen that even with a limited number of elements, the numerical solutions demonstrate high accuracy and rapid convergence. As the length scale parameter *l* increases, the natural frequency also increases, clearly demonstrating the size effect.

## 5. Conclusions

By analyzing the size-dependent axisymmetric deformation of small-scale solids with the 2D axisymmetric element, significant savings in computational costs can be achieved compared to using the 3D hexahedral element. In the present element formulation, the penalty function method is employed to address the *C*^1^ requirement for displacement, while the rotation field is approximated using independent nodal DOF. Consequently, the displacement test function can be simply designed using the enriched 4-node isoparametric shape function. Additionally, the trial function for the symmetric part of the force-stress, which represents the constitutive stress in the CCST, is formulated based on the force-stress field, which can a priori satisfy the relevant equilibrium equations for the concerned axisymmetric problem. Consequently, the general framework for the unsymmetric finite element method based on consistent couple stress theory for axisymmetric problems is proposed, specifically constructing a 4-node 12-DOF axisymmetric element. The following conclusions have been drawn:

(1) Numerical results indicate that the element demonstrates high computational accuracy and an excellent convergence rate in both static and modal analysis problems. It effectively captures size-dependent phenomena, showing a remarkable ability to represent the intricate behaviors of materials as their dimensions change. This performance highlights the element’s versatility and robustness, making it suitable for a wide range of applications in engineering and materials science. Furthermore, the consistency of results across various test cases underscores its reliability in predicting mechanical behavior in micro- and nanoscale structures.

(2) The new element incorporates only three conventional DOFs per node, facilitating its seamless integration into existing finite element programs or software. This simplicity not only enhances usability but also ensures that users can easily adopt the element without extensive modifications to their current systems. Such compatibility promotes efficiency in simulations and allows for rapid implementation in various engineering applications.

(3) The newly developed axisymmetric element, derived from the principle of virtual work, demonstrates a high degree of adaptability, making it well-suited for applications involving geometric nonlinearity. This flexibility allows for a more comprehensive analysis of complex structural behaviors under various loading conditions. The details of these related studies, including specific methodologies and results, will be thoroughly addressed in our forthcoming publications, where we aim to provide deeper insights into the performance and applicability of the element in practical scenarios.

## Figures and Tables

**Table 1:** The force-stress solutions used for formulating force-stress trial function.

	*i*	1	2	3	4	5	6	7	8	9	10	11	12	13	14	15
$H_{i}$	$\sigma_{\left(rr\right)}^{i}$	1/*r*	1	*z*/*r*	*r*	*z*	0	0	0	0	0	*z^2^*/*r*	0	0	0	0
	$\sigma_{\left(\theta \theta \right)}^{i}$	0	1	0	2 *r*	*z*	0	0	0	0	1	0	0	0	−2 *r*	2 *z*
	$\sigma_{\left(zz\right)}^{i}$	0	0	0	0	0	1/*r*	1	*z*/*r*	0	0	0	*r*	*z*	*z^2^*/*r*	0
	$\sigma_{\left(rz\right)}^{i}$	0	0	0	0	0	0	0	−1	1/*r*	*z*/*r*	0	0	−*r*/2	−2 *z*	*z^2^*/*r*

**Table 2:** The deflection convergence at point A under uniformly distributed load for a circular plate with *t* = 10 μm.

Mesh	*l*/*t*	2 × 20	4 × 40	8 × 80	16 × 160	Ref [57]
$u_{zA}$(μm)	0.1	10.5290	10.9082	11.0807	11.1638	11.0727
	0.5	2.6634	2.7190	2.7504	2.7670	2.7602
	1	0.8811	0.8971	0.9061	0.9108	0.9108
	10	0.1256	0.1270	0.1275	0.1277	0.1276

**Table 3:** The convergence results of the hollow circular plate.

Mesh		20 × 50 × 50	40 × 50 × 50	40 × 100 × 100	Ref [57]
$u_{rA}$(μm)	*l/h* = 0.01	0.50819	0.50819	0.50819	0.50690
	*l/h* = 0.1	0.50669	0.50668	0.50668	0.50607
	*l/h* = 0.5	0.50194	0.50195	0.50195	0.50179
	*l/h* = 1	0.50070	0.50070	0.50070	0.50064
$\omega_{\theta A}$	*l/h* = 0.01	0.0051574	0.0051547	0.0051549	0.0051770
	*l/h* = 0.1	0.0062310	0.0062182	0.0062182	0.0062216
	*l/h* = 0.5	0.0074398	0.0074128	0.0074128	0.0074149
	*l/h* = 1	0.0075213	0.0074949	0.0074949	0.0074912

**Table 4:** The convergence results of displacement $u_{rE}$ and displacement $u_{zD}$ (×10^−3^ μm).

Mesh	*l*/*r*	30	100	400	1600	6400	Ref [57]
$u_{rE}$	0.01	0.34602	0.34644	0.34573	0.34550	0.34542	0.34553
	0.1	0.32079	0.32803	0.32649	0.32624	0.32617	0.32610
	0.25	0.26892	0.27249	0.27014	0.26953	0.26936	0.26940
	0.5	0.20588	0.20250	0.20015	0.19943	0.19923	0.19911
	0.75	0.17111	0.16623	0.16418	0.16354	0.16335	0.16304
	1	0.15227	0.14687	0.14500	0.14440	0.14423	0.14379
$u_{zD}$	0.01	1.3705	1.3273	1.3347	1.3357	1.3362	1.3366
	0.1	1.3629	1.2840	1.2944	1.2971	1.2977	1.2978
	0.25	1.2385	1.1788	1.1829	1.1840	1.1842	1.1847
	0.5	1.0729	1.0413	1.0437	1.0441	1.0441	1.0437
	0.75	0.9845	0.9694	0.9720	0.9723	0.9723	0.9711
	1	0.9364	0.9307	0.9336	0.9341	0.9342	0.9324

**Table 5:** Normalized first three nondimensional natural frequencies of the simply supported circular plate.

	Mode			Mesh			Ref [58]
		1 × 20	2 × 100	5 × 100	8 × 400	10 × 500	
*l*/*h* = 0	1	5.0498	5.0488	5.0488	5.0488	5.0488	5.050
	2	29.7880	29.7496	29.7450	29.7445	29.7445	29.796
	3	74.1342	73.8609	73.8285	73.8285	73.8238	74.148
*l*/*h* = 0.2	1	6.8566	6.8195	6.7607	6.7625	6.7611	6.761
	2	38.4032	38.1934	37.9038	37.9094	37.9024	37.956
	3	94.9912	94.3240	93.6106	93.6059	93.5874	93.963
*l*/*h* = 0.4	1	10.3400	10.3150	10.2765	10.2774	10.2765	10.279
	2	55.9088	55.7373	55.5428	55.5428	55.5381	55.654
	3	137.6037	136.9505	136.4641	136.4455	136.4316	137.182
*l*/*h* = 0.6	1	14.3663	14.3474	14.3196	14.3205	14.3196	14.328
	2	76.5943	76.4321	76.2885	76.2885	76.2839	76.539
	3	187.8790	187.1702	186.8181	186.7810	186.7718	188.328

**Table 6:** Normalized first three nondimensional natural frequencies of the clamped circular plate.

	Mode			Mesh			Ref [58]
		1 × 20	2 × 100	5 × 100	8 × 400	10 × 500	
*l*/*h* = 0	1	10.2126	10.2195	10.2177	10.2140	10.2135	10.215
	2	39.8009	39.6828	39.6689	39.6522	39.6504	39.753
	3	89.3438	88.5562	88.5052	88.4589	88.4543	88.987
*l*/*h* = 0.2	1	12.9714	13.0011	12.9228	12.9149	12.9121	12.901
	2	50.5440	50.4884	50.1780	50.1363	50.1270	50.206
	3	113.4066	112.6839	111.9704	111.8592	111.8361	112.392
*l*/*h* = 0.4	1	18.8231	18.8347	18.7823	18.7772	18.7754	18.782
	2	73.2957	73.0872	72.8741	72.8463	72.8417	73.087
	3	164.2887	162.9452	162.4634	162.3615	162.3476	163.622
*l*/*h* = 0.6	1	25.7631	25.7598	25.7214	25.7177	25.7163	25.754
	2	100.1753	99.8139	99.6610	99.6332	99.6286	100.217
	3	224.0798	222.0738	221.7124	221.6012	221.5873	224.362

## Data Availability

The data that support the findings of this study are available from the corresponding author upon reasonable request.
